# Association between Intestinal Colonization and Extraintestinal Infection with Carbapenem-Resistant Klebsiella pneumoniae in Children

**DOI:** 10.1128/spectrum.04088-22

**Published:** 2023-03-14

**Authors:** Qingqing Du, Qi Xu, Fen Pan, Yingying Shi, Fangyuan Yu, Tiandong Zhang, Jie Jiang, Wenxin Liu, Xiaozhou Pan, Dingding Han, Hong Zhang

**Affiliations:** a Department of Clinical Laboratory, Shanghai Children’s Hospital, School of Medicine, Shanghai Jiao Tong University, Shanghai, China; b Institute of Pediatric Infection, Immunity, and Critical Care Medicine, Shanghai Jiao Tong University School of Medicine, Shanghai, China; c Department of Infectious Diseases, Research Laboratory of Clinical Virology, Ruijin Hospital, Shanghai Jiao Tong University, School of Medicine, Shanghai, China; Yangzhou University

**Keywords:** *Klebsiella pneumoniae*, carbapenem resistance, gastrointestinal colonization, whole-genome sequencing, hospital-acquired infection

## Abstract

Carbapenem-resistant Klebsiella pneumoniae (CRKP) has become a critical public health threat. However, the association between intestinal colonization and parenteral infection among pediatric patients has not been elucidated. We collected 8 fecal CRKP strains and 10 corresponding CRKP strains responsible for extraintestinal infection from eight patients who did not manifest infection upon admission to the hospital. Paired isolates showed identical resistance to antimicrobials and identical virulence *in vitro* and *in vivo*. *wzi* capsule typing, multilocus sequence typing, and whole-genome sequencing (WGS) indicated high similarity between paired colonizing and infecting isolates. Mutations between colonizing and infecting isolate pairs found by WGS had a distinctive molecular signature of a high proportion of complex structural variants. The mutated genes were involved in pathways associated with infection-related physiological and pathogenic functions, including antibiotic resistance, virulence, and response to the extracellular environment. The latter is important for bacterial infection of environmental niches. Various mutations related to antibiotic resistance, virulence, and colonization that were not associated with any particular mutational hot spot correlated with an increased risk of extraintestinal infection. Notably, novel subclone carbapenem-resistant hypervirulent K. pneumoniae (CR-hvKP) KL19-ST15 exhibited hypervirulence in experimental assays that reflected the severe clinical symptoms of two patients infected with the clonal strains. Taken together, our findings indicate the association between CRKP intestinal colonization and extraintestinal infection, suggesting that active screening for colonization on admission could decrease infection risk in children.

**IMPORTANCE** Carbapenem-resistant Klebsiella pneumoniae (CRKP) causes an increasing number of nosocomial infections, which can be life-threatening, as carbapenems are last-resort antibiotics. K. pneumoniae is part of the healthy human microbiome, and this provides a potential advantage for infection. This study demonstrated that CRKP intestinal colonization is strongly linked to extraintestinal infection, based on the evidence given by whole-genome sequencing data and phenotypic assays of antimicrobial resistance and virulence. Apart from these findings, our in-depth analysis of point mutations and chromosome structural variants in patient-specific infecting isolates compared with colonizing isolates may contribute insights into bacterial adaptation underlying CRKP infection. In addition, a novel subclone of carbapenem-resistant hypervirulent K. pneumoniae (CR-hvKP) was observed in the study. This finding highlights the importance of CRKP active surveillance among children, targeting in particular the novel high-risk CR-hvKP clone.

## INTRODUCTION

Carbapenem-resistant Klebsiella pneumoniae (CRKP) has emerged as a leading cause of nosocomial infection and is associated with high rates of morbidity and mortality due to the dearth of therapeutic options (1, 2). Antibiotic resistance is more problematic in children than in adults because of differences in carbapenemase production among strains, as children tend to become infected with a mixture of NDM, KPC, and OXA-48–like producers, in contrast to the overwhelming prevalence of KPC-2–producing strains in adult patients (3). Thus, it can be difficult to track the dissemination of CRKP strains and control these infections. Furthermore, hypervirulent K. pneumoniae strains can cause life-threatening complications, such as liver abscesses, severe pneumonia, and endophthalmitis, in healthy individuals because such strains exhibit excessive capsule production and iron acquisition (4). Therefore, the convergence of carbapenem resistance and hypervirulence leads to high rates of patient mortality (5). Infection with carbapenem-resistant hypervirulent K. pneumoniae (CR-hvKP) has been increasingly reported (6, 7), suggesting that these strains can spread readily in clinical settings and cause fatal outbreaks (1). Therefore, CR-hvKP has the potential to be the next “superbug” posing a threat to immunocompromised pediatric patients.

K. pneumoniae naturally colonizes the human nasopharyngeal and gastrointestinal tracts, and the rate of gastrointestinal tract colonization is typically higher (8). In addition, K. pneumoniae can cause serious infections, including pneumonia, urinary tract infections, and bloodstream infections (9). Importantly, several recent studies have demonstrated that gastrointestinal colonization with K. pneumoniae upon admission to the hospital is a common and significant risk factor of transmission to and subsequent infection of adult patients in the intensive care unit (10–12). However, the association between intestinal colonization and extraintestinal infection among pediatric patients has not been well explored. At our hospital, we have implemented active screening of patients before admission via culture surveillance to allow early identification of CRKP colonization and adoption of infection control measures (such as contact isolation) to reduce the spread of the pathogen (13).

While previous studies have performed homology analysis of patient-specific paired colonizing and infecting isolates, in this study we applied whole-genome sequencing (WGS) to perform an in-depth analysis of point mutations and other mutational variants that arose within individual patients infected with CRKP to gain a better understanding of the bacterial adaptations that underlie persistent infection. Furthermore, we characterized the virulence and antibiotic resistance of the CRKP isolates at both the genotype and phenotype levels to evaluate whether the strains associated with extraintestinal infection exhibited the same profiles as the strains associated with colonization.

## RESULTS

### Identification of patients with CRKP intestinal colonization and extraintestinal infection by epidemiological surveillance.

**FIG 1:**
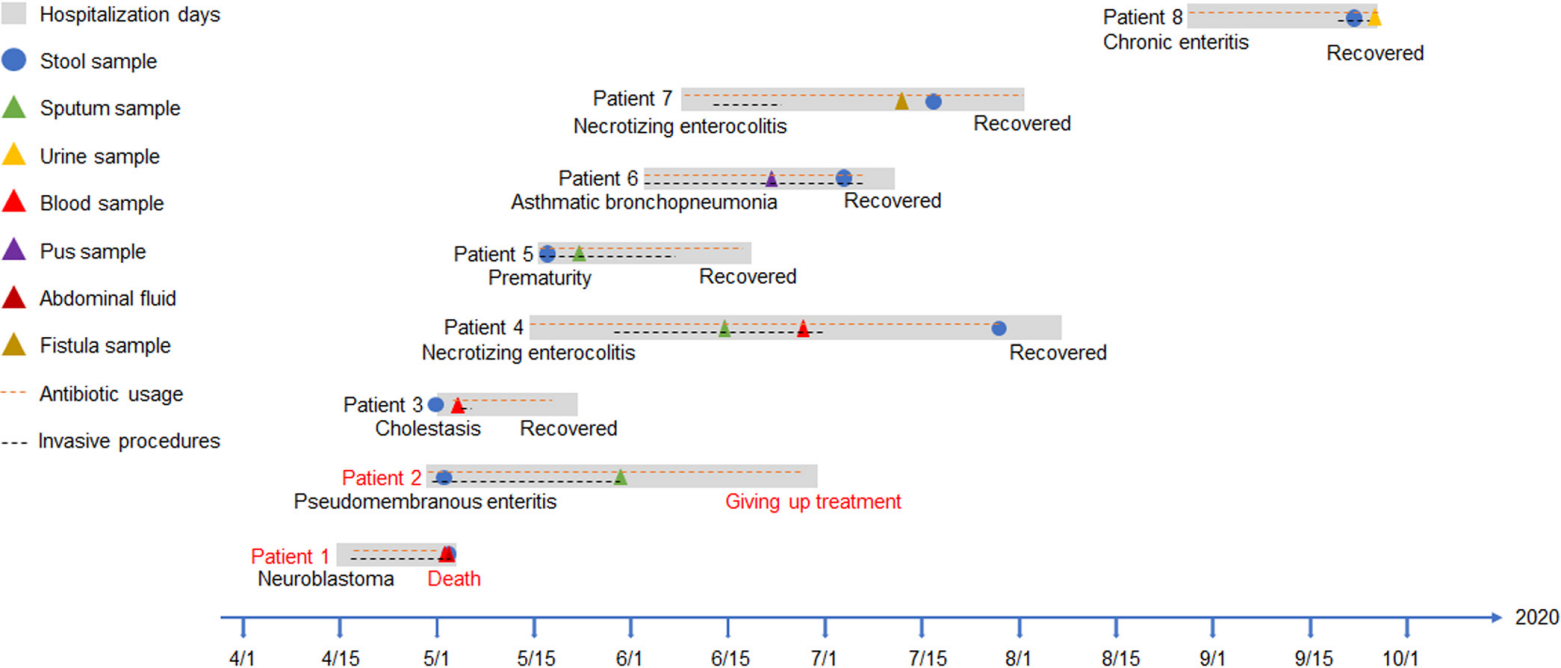
Timeline of isolation of CRKP strains from eight pediatric patients. Triangles indicate infecting CRKP isolates; circles indicate fecal isolates. Red text indicates poor patient outcome.

Eight patients who were intestinally colonized with CRKP were also identified as suffering from extraintestinal infection between April and October 2020, including 4 neonatal patients (4 males), 3 pediatric intensive care unit (PICU) patients (3 females), and 1 gastroenterology patient (1 female). The age of infection onset ranged from 1 to 3 months in neonatal patients and 1 to 7 years in nonneonatal patients. Eighteen CRKP strains were recovered from these eight patients, including 8 distinctive colonizing strains isolated from fecal swabs and 10 distinctive infecting strains isolated from various specimens, including sputum, urine, blood, pus, abdominal fluid, and fistula fluid. Paired infecting strains from patients 1, 2, 3, 5, and 8 were isolated after colonization, while other paired infecting strains, isolated from patients 4, 6, and 7, were isolated before CRKP colonization active screening. During hospitalization, all of these patients underwent antimicrobial treatment and invasive procedures (e.g., mechanical ventilation or surgery) (Fig. 1). In addition, nine CRKP infecting strains were isolated from nine patients who were only infected with CRKP but without intestinal colonization, and five CRKP colonizing strains were isolated from five patients who were identified as only colonized with CRKP but without extraintestinal infection (see Table S2 in the supplemental material).

**FIG 2:**
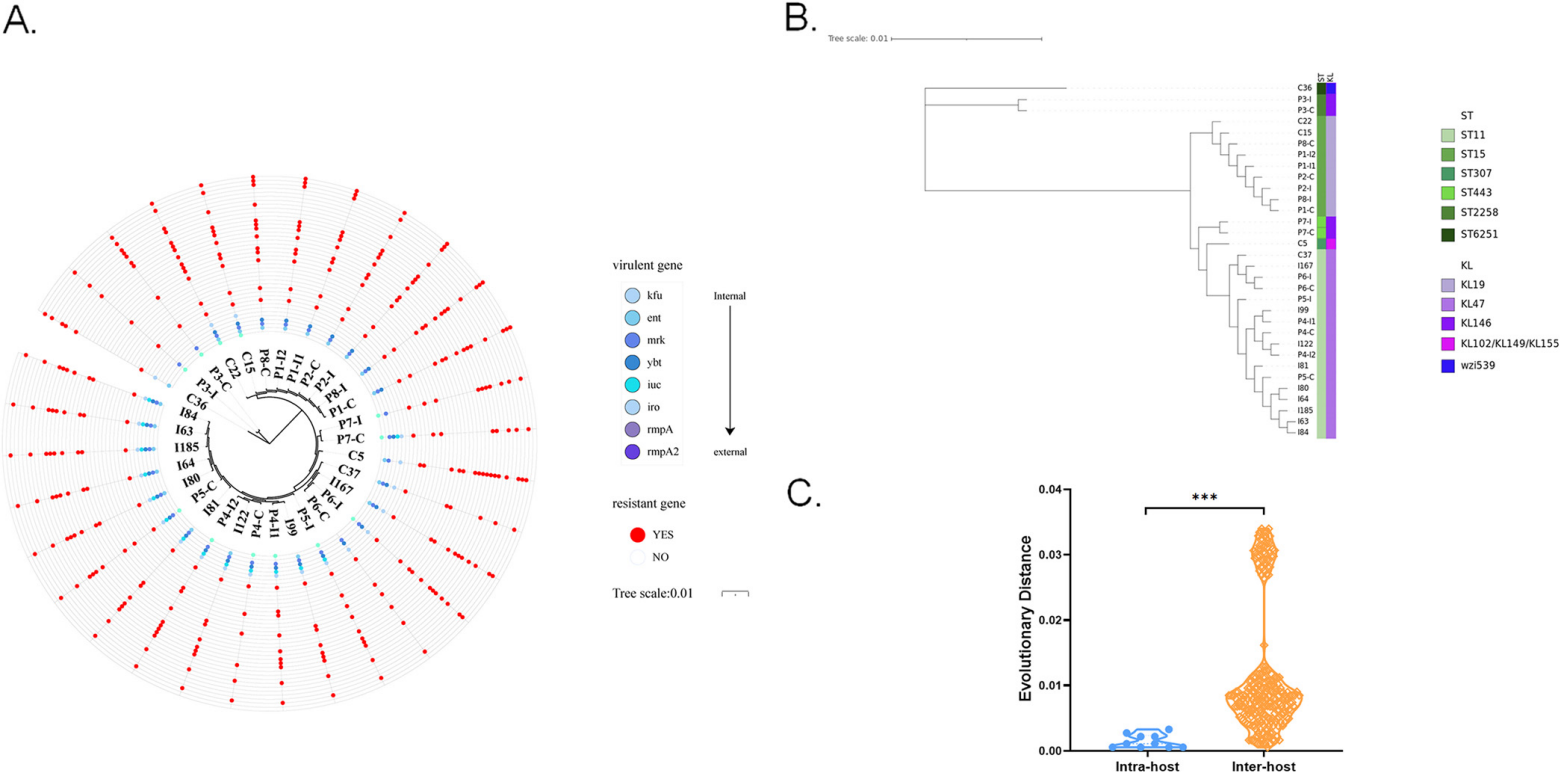
Phylogenetic analysis of paired colonizing and infecting isolates, independent colonizing isolates (without infection), and independent infecting isolates (without colonization). (A and B) A global tree (A) and a local tree (B) were constructed by consensus CRKP genomes using FastTree with the maximum-likelihood algorithm. In the resistant genes panel, genes from inside to outside were *bla*_KPC-2_, *bla*_NDM-1_, *bla*_IMP-4_, *bla*_OXA-1_, *bla*_SHV-1_, *bla*_SHV-1a_, *bla*_SHV-2a_, *bla*_SHV-11_, *bla*_SHV-12_, *bla*_SHV-25_, *bla*_SHV-28_, *bla*_SHV-66_, *bla*_TEM-1b_, *bla*_CTX-M-15_, *bla*_CTX-M-18_, *bla*_CTX-M-27_, *bla*_CTX-M-65_, *tet*(A), *tetR*, *tetG*, *tet34*, *sul1*, *sul2*, *qnrS1*, *dfrA3*, *dfrA5*, *dfrA12*, *dfrA14*, *aph*(6)*-ld*, *aph*(3‴)*-lb*, *aac*(6′)*-lb-cr*, and *aac*(3)*-lla*. Strains I63, I64, I80, I81, I84, I99, I122, I167, and I185 were isolated from 9 patients who were infected with CRKP but without intestinal colonization; strains C5, C15, C22, C36, and C37 were isolated from 5 patients who were colonized with CRKP but without extraintestinal infection; strains P1-C, P1-I1, P1-I2, P2-C, P2-I, P3-C, P3-I, P4-I1, P4-I2, P5-C, P5-I, P6-C, P6-I, P7-C, P7-I, P8-C, and P8-I were isolated from 8 patients who had both intestinal colonization and extraintestinal infection. P, patient; C, colonizing isolate; I, infecting isolate. (C) Genetic distance between colonizing and infecting isolates in intrahost group and interhost group analyses, shown by violin plots. Each dot represents the genetic distance between a unique pair of colonizing and infecting isolates. Genetic distances among paired isolates from the same patient were significantly smaller than those among interhost isolates (*P < *0.0001).

By WGS and PCR validation, *bla*_KPC-2_ (87.5%, 28/32) was found to be the most prevalent carbapenemase-encoding gene, followed by *bla*_NDM-1_ (9.4%, 3/32) and *bla*_IMP-4_ (3.1%, 1/32). All isolates carried extended-spectrum β-lactamase (ESBL) genes, with substantially higher rates for *bla*_TEM-1_ (78.1%, 25/32) and *bla*_CTX-M-65_ (71.9%, 23/32). The positive rates of additional tested resistance genes varied substantially, ranging from 3.1% to 100% (Fig. 2A). Regarding virulence-associated genes, we found that nearly all the CRKP isolates carried *mrk* (96.9%, 31/32), and fewer carried *kfu* (37.5%, 12/32). The hypervirulence determinants *rmpA* and *rmpA2* were detected in 6 and 13 isolates by PCR, but their function may be interrupted by nonsense mutations inferred by WGS. Nevertheless, consistent results from PCR and WGS were obtained for the other virulence genes, including *iuc* (43.8%, 14/32) and *iro* (68.8%, 22/32) (Fig. 2A). Of the 32 CRKP strains, 53.1% (17/32) were the KL47-ST11 clone, followed by the KL19-ST15 clone (28.1%, 9/32) (Fig. 2B).

### Intestinal colonization with CRKP is linked to extraintestinal infection revealed by WGS.

To evaluate the association among the intrahost isolates and the interpatient isolates, we evaluated genetic clonal concordance by WGS among 32 isolates from 26 patients, including paired CRKP colonizing and infecting isolates from 8 patients, independent CRKP colonizing isolates from 5 patients (without infection), and independent CRKP infecting isolates from 9 patients (without colonization). All of the 32 strains shared a total of 3,821 common gene families, among which only a few gene families were unique to each pair of CRKP carriage and infection isolates from eight patients by the genome comparison (Fig. S1). According to the core genome phylogenetic tree, five of eight pairs (62.5%) matched at the lineage level (including three patients whose infecting isolate was collected prior to carriage) (Fig. 2B). Genetic distances among paired colonizing and infecting isolates from the same patient were significantly smaller than those among interhost isolates (*P < *0.0001) (Fig. 2C). To validate *wzi* typing and multilocus sequence typing (MLST), we further performed enterobacterial repetitive intergenic consensus PRC (ERIC- PCR) analysis. The fingerprint results showed consistency between paired colonizing and infecting isolates (Fig. S2). Taken together, the capsule serotype, MLST, ERIC-PCR pattern, and phylogenetic analysis results indicated that the infecting isolates were clonally related to the colonizing strains at the molecular level.

### *In vitro* and *in vivo* antimicrobial resistance and virulence assessment of the paired CRKP isolates.

**FIG 3:**
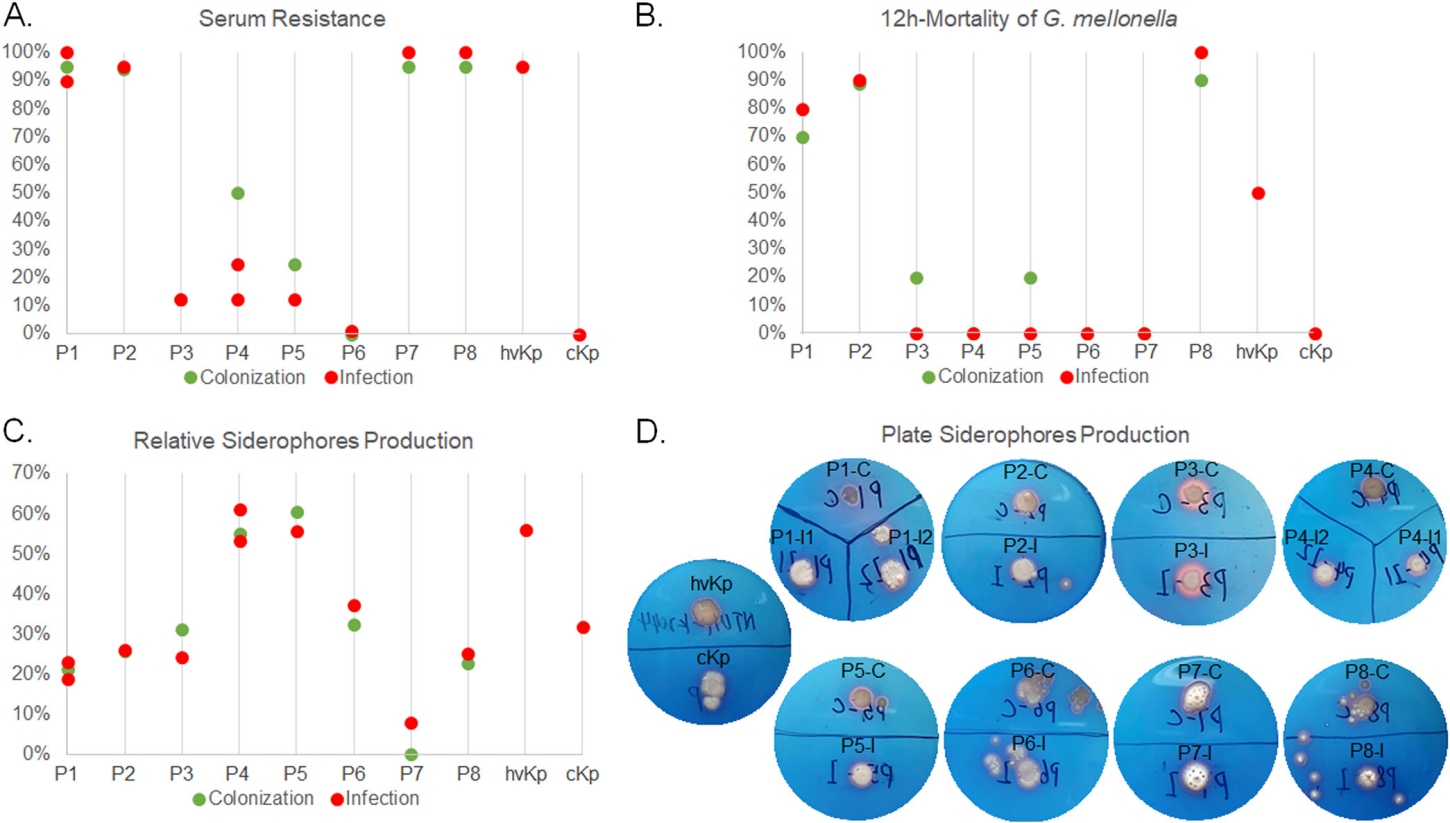
Virulence assessment of the paired colonizing and infecting CRKP strains from eight patients, as well as reference strains. Results are shown for serum resistance (A), 12-h induction of Galleria mellonella mortality (B), relative siderophore production (C), and plate-based siderophore production (D) of the experimental and reference strains. Abbreviations: P1 to P8, patients 1 to 8; C, colonization; I, infection.

We tested 17 antimicrobials for their activity against the isolates, and measured categorical agreement (CA) for susceptible, intermediate, or resistant phenotypes. The CA for antimicrobial susceptibility was as high as 100% for eight pairs of colonizing and infecting isolates (Fig. S3). In addition to the resistance profiles, paired colonizing and infecting isolates demonstrated high concordance in terms of virulence phenotype (Fig. 3). For resistance to human serum, lethality to Galleria mellonella, and quantitative siderophore production (SP), the paired strains had intraclass correlation coefficients (ICCs) of 0.966 (*P < *0.001), 0.971 (*P < *0.001), and 0.984 (*P < *0.001), respectively.

Notably, patient 1 (P1) and patient 2 (P2) died from CRKP septic shock and had a risk of death due to severe pulmonary infection, respectively, which were considerably worse outcomes than those of the other six patients (Fig. 1 and Table S2). We therefore compared the virulence potential between five isolates from P1 and P2 and 13 isolates from other patients. The string test was negative for all 18 CRKP strains. In the presence of human serum, nine CRKP isolates from P1, P2, P7, and P8 exhibited >95% survival, which was similar to the rates seen with the hypervirulent NTUH-K2044 strain but significantly higher than that seen with isolates from P3, P4, P5, P6, P9, and P10 and with classical K. pneumoniae (cKp) (*P < 0.001*) (Fig. 3A). The G. mellonella infection results showed that the mortality of larvae infected by six isolates from P1, P2, and P8 was >50%, while the lethality of those infected with the other 12 isolates was <20% at 12 h after infection (the survival curves remained stable after 12 h), suggesting that the isolates from P1, P2, and P8 were more virulent than the other isolates (Fig. 3B). Interestingly, isolates from these patients were grouped into one cluster in the phylogenetic tree. Furthermore, paired strains from P4 and P5 showed similar siderophore production as that of NTUH-K2044 in the solution-based assay (Fig. 3C), indicating that the putative aerobactin and salmochelin genes exerted their normal function. In the qualitative SP plate assay, all pairs of colonizing and infecting isolates developed an orange halo after incubation, except for the cKp control (Fig. 3D). The strains that exhibited carbapenem resistance and hypervirulence from P1, P2, and P8 were identified as KL19-ST15 CR-hvKP, which is a novel CR-hvKP clone rarely reported until 2022 (14).

### Genetic adaption of the intrahost isolates.

**FIG 4:**
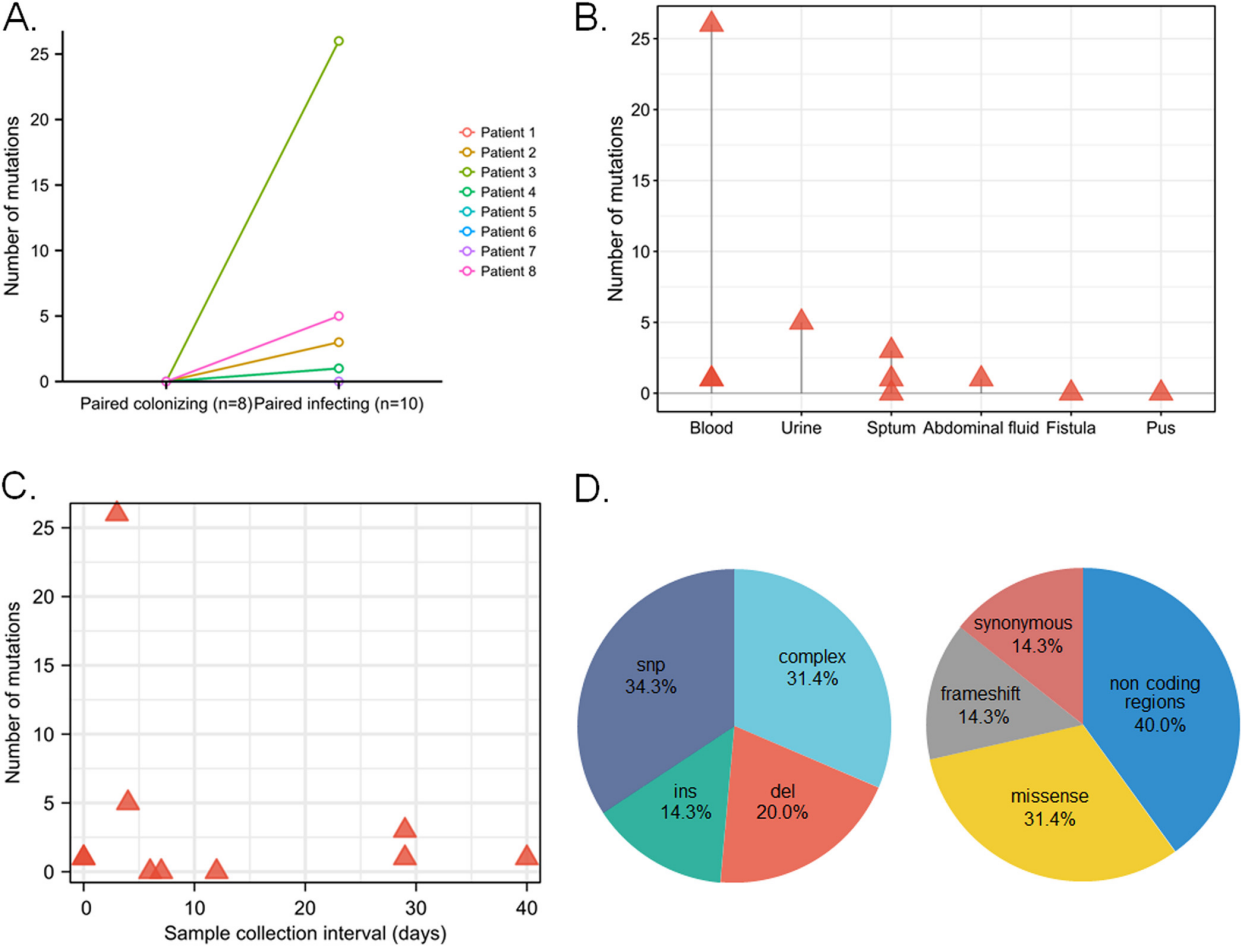
Variants identified by episode-specific mapping and variant calling. (A) Number of mutations in each pair of colonizing and infecting isolates from each patient. (B) Number of mutations in infecting isolates according to sample type. (C) Correlation between sample collection interval and number of mutations. (D) Distribution of mutation types in infecting isolates compared with their corresponding colonizing isolates. Abbreviations: snp, single-nucleotide polymorphism; ins, insertion; del, deletion.

To determine the variants that emerged in infecting isolates under host immune pressure, reads were mapped to both the reference genome and to the *de novo* assembly of the corresponding colonizing strains in a patient-specific manner. A total of 38 variants were identified in the 8 patients, including 35 unique variants. At least one mutation was found in 5 out of 8 episodes (Fig. 4A), mainly in strains isolated from blood, urine, and sputum samples (Fig. 4B). No association was found between the number of mutations and the time interval between collection of the paired isolates (either from colonization to infection or infection to colonization) (Fig. 4C).

**FIG 5:**
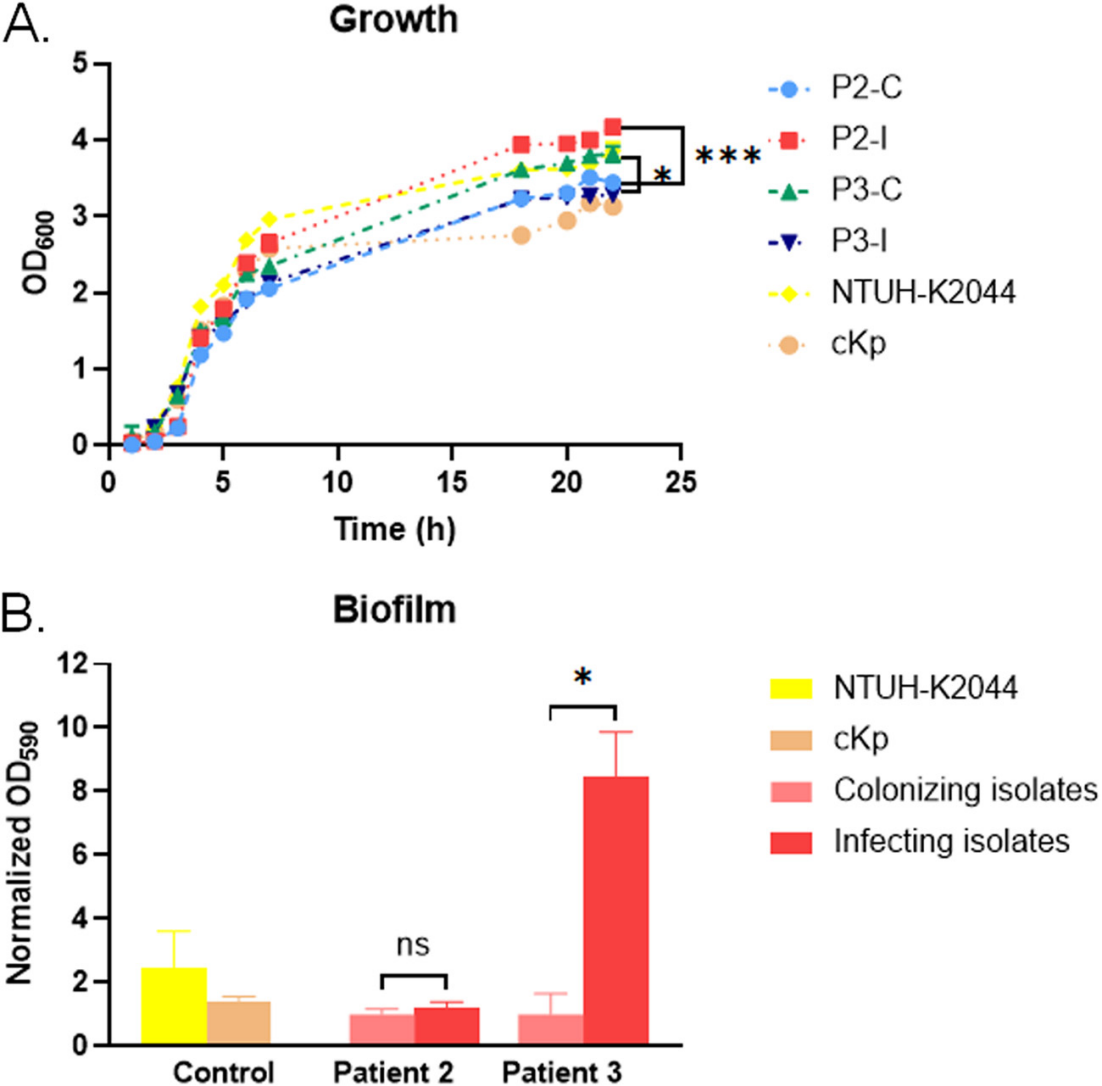
Phenotypic changes with mutations in paired colonizing and infecting isolates from patient 2 and patient 3. (A) Growth curves of selected paired isolates and control K. pneumoniae isolates. There was a significant difference in growth between colonizing isolate and paired infecting isolate from patient 2 (*P < *0.0001) and patient 3 (*P = *0.0115). (B) Analysis of biofilm formation, based on normalized OD_590_ (calculated as the OD_590_ of the paired colonizing and infecting isolates from each patient divided by the OD_590_ of the corresponding colonizing isolate from each patient). There was a significant difference in biofilm production between colonizing isolate and paired infecting isolate in patient 3 (*P = *0.0231), but no significance between that from patient 2 (*P = *0.2758). Abbreviations: P1 to P8: patients 1 to 8; C, colonization; I, infection. Data shown are means ± standard deviations. *, *P < *0.05; ***, *P < *0.001; ns, *P > *0.05.

The unique variants included 11 complex variants, 12 single-nucleotide polymorphisms, and 12 indels (Fig. 4D). Among these variants, 16 (16/35, 45.7%) were predicted to affect protein function: 11 were missense substitutions and 5 were frameshift mutations (Table S3). Interestingly, the frameshift mutations were all detected in patient 3. To investigate whether the predicted genetic variants would cause phenotypic alterations, we selected paired isolates from P2 and P3, and the functions of the mutant genes they carried have been well studied. For example, the mutant gene *KPHS_42880* in P2 was predicted to affect growth, while the mutant gene *KPHS_19380* in P3 may affect biofilm formation. Indeed, the growth rate of the infecting isolate from P2 significantly increased above that of the paired colonizing isolate (*P < *0.0001) (Fig. 5A). Besides, the infecting isolate from P3 formed more biofilm than that in the paired colonizing isolate (*P = *0.0231), while the paired infecting and colonizing isolates from P2 showed no significant difference in biofilm production (*P = *0.2758) (Fig. 5B).

## DISCUSSION

We observed a high concordance between intestinal colonizing and corresponding extraintestinal infecting CRKP strains in eight pediatric patients, based on *wzi* typing, MLST, and WGS data, as well as antimicrobial resistance and virulence patterns, suggesting that colonization is crucial for developing hospital-acquired infections. Nonetheless, the isolates P5-C and P5-I were evolutionarily distant. Between the isolates, there were five infecting strains isolated from newborns in the neonatology ward, suggesting that P5-I strain was probably acquired from the intrahospital dissemination. However, nine independent infecting isolates (without colonization) and five independent colonizing isolates (without infection) were clustered separately, in which none of the colonizing CRKP and infecting CRKP isolates from different patients was combined in pairs. Paired colonizing and infecting isolates from the same patient had significantly smaller genetic distances than interhost isolates (*P < *0.0001), suggesting the intrinsic association of the paired colonizing and infecting isolates from the same individuals.

According to the MLST results, the most dominant sequence types of the 18 clonally related pairs of colonizing-infecting CRKP strains isolated from 8 patients were ST15 (38.9%, 7/18) and ST11 (38.9%, 7/18). ST11 is a single-locus variant of ST258 and is reported to be the most prevalent type of CRKP in China, while the prevalence of ST15 has increased drastically in recent years (15). In this study, KL19-ST15 CR-hvKP strains were isolated from patients 1, 2, and 8 in the PICU and the gastroenterology department, and both colonizing and infecting KL19-ST15 isolates exhibited high resistance to human serum, high mortality for G. mellonella, and caused fatal infections in some of our pediatric patients (Fig. 3 and Table S2). Considering the hospitalization duration between patient 1 and patient 8, the CR-hvKP clone may have been transmitted within the hospital for at least 6 months (Fig. 1). Notably, the convergence of carbapenem resistance and hypervirulence led to a high mortality rate among the children included in our study. Hence, controlling nosocomial CR-hvKP outbreaks requires early identification of patients carrying the clonal strains. We found that the KL19 serotype was strongly associated with serum resistance and G. mellonella killing, as reported previously (16). In this study, the results from the qualitative siderophore production solution assay and the qualitative plate assay were not always consistent, reflecting these tests’ different capacities for identifying hvKP strains (17). Cooccurrence of the *iuc* and *iro* loci was found in two pairs of strains from patients 4 and 5, which might explain the excessive production of siderophores by these strains.

By analyzing intrahost variation between paired colonizing and infecting isolates, we found that most infecting isolates had a limited number of mutations. Surprisingly, unlike isolates from other blood samples that contained only one mutation, the infecting isolate from patient 3 exhibited 26 mutations, even though the time interval from colonization to infection was only 3 days. This may reflect the bacteria’s active response to different host immune stresses. Mutations were found in five out of eight pairs of isolates, and over one-third of these mutations were complex structural variants. Complex structural variants generally occur rarely, because of the requirement for two or more breakpoints (18). The high frequency of this type of variant observed in this study suggests instability during transition between colonization and infection (19). The most common mutation types that we observed were missense point mutations in coding and noncoding regions.

A similar proportion of mutation types was also observed between colonizing and infecting isolates in Staphylococcus aureus bacteremia, suggesting that this may be a general rule for bacteria (20). In total, we identified nonsilent mutations in 16 annotated genes in 10 infecting isolates. These genes participate in a broad spectrum of signaling pathways. Nevertheless, their physiological and pathogenic functions in bacteria converged on a few infection-related aspects, including antibiotic resistance, virulence, and response to the extracellular environment. The latter is important for bacterial infection of environmental niches. The gene *KPHS_19380* encodes a glucans biosynthesis protein, family members of which are involved in biofilm formation, virulence, and resistance to antibiotics (21). Inactivation of these genes may result in increased expression of colanic acid, which is required for normal biofilm formation (22). In addition to frameshift mutations, missense mutations may also influence organisms’ physiology and pathology. The gene *KPHS_42880* encodes *N*-acetylmuramoyl-l-alanine amidase, which plays a role in cleaving intact peptidoglycan during cell division and exhibits autolytic activity in the presence of antibiotics. A previous study reported that overexpression of this enzyme resulted in lysis and hypersensitivity to low levels of antibiotics. *KPHS_42880* deletion mutants display tolerance to antibiotics (23). Adenine deaminase is believed to convert adenine to GMP and is involved in bacterial nitrogen metabolism (24). Thus, mutations in *KPHS_04830*, which encodes adenine deaminase dihydroorotase, may affect bacterial function and growth. *KPHS_42440*, which encodes the tRNA cyclic N^6^-threonylcarbamoyladenosine synthase TcdA (20), plays a crucial role in maintaining tRNA decoding accuracy and controlling gene expression (25). Moreover, *KPHS_48410* encodes a zinc-responsive transcriptional regulator that has been implicated in zinc homeostasis and bacterial virulence (26).

The gene *KPHS_23180* encodes type VI secretion system baseplate subunit TssG. The type VI secretion system is a specialized secretion system of Gram-negative bacteria that exhibits antibacterial activity toward neighboring bacterial cells in a contact-dependent manner and is also involved in biofilm formation, antibiotic resistance, and host adaptation (27). The gene *KPHS_28360* encodes the formate dehydrogenase-N alpha subunit. Under anaerobic cell growth conditions, formate dehydrogenase can oxidize formate to CO_2_ and pass electrons to either the quinone pool or other proteins for cellular energy conservation and ATP generation (28). However, the role of bacterial formate dehydrogenase in colonization and infection has not been explored. The gene *KPHS_52510* encodes a DNA-binding transcriptional regulator called DsdC, which is a d-serine-dependent activator of *dsdXA* transcription. A DsdC mutant strain showed hypervirulence in a murine model (29). Meropenem has been reported to show low activity against K. pneumoniae isolates carrying the gene *KPHS_46730* (30). Thus, frameshift mutations in *KPHS_46730* may convey resistance to meropenem. In addition, mutations in *KPHS_11630*, which encodes a GntR family transcriptional regulator, may affect a variety of cellular processes, such as cell motility, glucose metabolism, bacterial resistance, pathogenesis, and virulence (31).

Apart from these coding sequence mutations, we found four mutations in the promoter regions that may affect transcription and expression of the corresponding genes. First, *KPHS_05940* encodes an antibiotic transport system permease component and functions in bacterial self-immunity, antibiotic transport, and antibiotic resistance (32). Second, *KPHS_10490*, *KPHS_10500*, and *KPHS_10510* encode taurine transporters that are involved in scavenging sulfur to promote bacterial growth during colonization (33). Third, *KPHS_31760* encodes the type V toxin-antitoxin system endoribonuclease antitoxin GhoS, which inhibits the toxin by specifically cleaving its mRNA, suggesting that it plays a crucial role in multidrug tolerance and biofilm formation (34). Fourth, *KPHS_47580* encodes the aerobic respiration control sensor protein ArcB, which regulates anaerobic repression to facilitate bacterial respiratory conditions and growth (35). In this study, we found that isolates with a cell division-related mutation from P2 substantially influenced the growth rate, while isolates with a biofilm-related mutation from P3 showed drastic changes in biofilm formation. These findings may reflect the different strategies used by the bacteria to adapt to the host and environmental stresses. However, many of the genes in which mutations were found in this study have not been well characterized. Additionally, we only assessed a limited array of phenotypes. Therefore, the effects of these mutations require further study. In-depth investigations of these genes should be carried out to explore the mechanisms involved in the transition between CRKP colonization and infection.

One study reported that mortality was higher in patients colonized with multidrug-resistant organisms (36). Yet, decolonization is a controversial treatment approach, as selective digestive decontamination is also associated with an increase in antibiotic resistance (37). In any case, active surveillance and appropriate contact isolation are conducive to decreasing CRKP colonization and infection. The limited sample size also makes it difficult to draw conclusions regarding the functional adaptations of the CRKP strains associated with the identified mutations. Furthermore, we did not clarify the detailed pathogenetic pathway from colonization to infection in the present study.

### Conclusion.

In conclusion, our study revealed that colonizing CRKP may act as a reservoir for infection, which suggests that surveillance could help prevent nosocomial infection outbreaks. Additionally, we found that a novel CR-hvKP subclone, KL19-ST15, led to poor outcomes in infected patients because of its hypervirulence, as determined by *in vitro* and *in vivo* assays. Finally, genomic comparison of eight pairs of colonizing and infecting CRKP isolates suggested that the genetic alternations that we observed in the infecting CRKP strains may have been induced by the combined pressure of antibiotics and host immunity. These findings will improve our understanding of the pathogenesis of CRKP and contribute to nosocomial infection management.

## MATERIALS AND METHODS

### Isolates and patients.

To decrease the incidence of carbapenem-resistant *Enterobacterales* (CRE) nosocomial infections, screening for intestinal and upper respiratory tract CRE colonization in patients has been conducted at Shanghai Children’s Hospital (Shanghai, China) according to the WHO Guidelines (38). Strains isolated from rectal swab or stool samples of inpatients without intestinal bacterial infection manifestations were defined as colonizing strains and, correspondingly, strains isolated from extraintestinal specimens (e.g., blood, sputum, urine, pus, fistula, and abdominal fluid) of inpatients with associated infection manifestations were defined as infecting strains (39, 40). The species identity of all of the isolates was confirmed by matrix-assisted laser desorption ionization–time-of-flight mass spectrometry (Bruker Daltonik GmbH, Bremen, Germany). K. pneumoniae was identified as CRKP by its resistance to one of the following: ertapenem, imipenem or meropenem.

Based on active screening from March to December 2020, we collected paired colonizing and infecting isolates from eight patients. In addition, during the period, we also collected isolates from nine patients who were only infected with CRKP but without intestinal colonization and from five patients who were only colonized with CRKP but without extraintestinal infection.

### Ethics statement.

Ethical approval for this study was granted by the Shanghai Children’s Hospital Ethics Review Committee (approval number 2022R123-E01).

### Antimicrobial susceptibility testing of CRKP isolates.

The MICs of 17 antimicrobial agents, including cefotaxime, ceftazidime, cefepime, ciprofloxacin, piperacillin-tazobactam, cefoperazone-sulbactam, trimethoprim-sulfamethoxazole, ceftazidime-avibactam, amikacin, gentamicin, ertapenem, imipenem, meropenem, aztreonam, fosfomycin, colistin, and tigecycline, were examined using the broth microdilution method. The results were interpreted according to Clinical and Laboratory Standards Institute guidelines (41). Breakpoints for tigecycline were as defined by the U.S. Food and Drug Administration (42). Quality controls for susceptibility testing were performed with Escherichia coli ATCC 25922. Initial screening for ESBL was accomplished using the Vitek 2 Advanced Expert system (bioMérieux) according to the manufacturer's instructions.

### Whole-genome sequencing and *de novo* assembly.

Whole-genome sequencing was carried out using an Illumina NovaSeq sequencing platform (Illumina Inc., San Diego, CA) with a 2 × 150-bp read length (Biozeron Biotechnology, Shanghai, China). Clean reads were obtained after removing the adapter sequences and low-quality sequences. *De novo* assembly of the clean data was performed using A5-Miseq v20160825 (43) and SPAdes v3.12.0 (44), and base correction for the assembly was performed with Pilon v1.18 (45).

### *In silico* identification of ST, capsular type, virulence genes, and resistance genes.

WGS data were used for genotypic characterization, including the determination of the sequence type (ST) and capsular typing (*wzi* sequencing) with Institut Pasteur online tools (https://bigsdb.pasteur.fr/klebsiella/) (46). Virulence genes were identified using the virulence factor database (VFDB) (47), and resistance genes were identified using the comprehensive antibiotic resistance database (CARD) (https://card.mcmaster.ca/) and the Resfinder database (https://cge.cbs.dtu.dk/services/).

### PCR and Sanger sequencing for validation of resistance genes, virulence gene ST, capsular type, and visualized homology analysis.

Standard PCR and Sanger sequencing were performed to validate carbapenemase genes, ESBL genes, and virulence genes using specific primers (listed in Table S1). Capsule serotyping and MLST determinations were carried out by detecting one capsular serotype-specific gene (*wzi*) and seven housekeeping genes (*ropB*, *gapA*, *mdh*, *pgi*, *phoE*, *infB*, and *tonB*), respectively (Table S1). Then, the sequences were submitted to the Institute Pasteur database to determine ST. ERIC-PCR was performed using gene-specific primers (listed in Table S1), and the patterns were analyzed using BioNumerics software (Applied Maths; NV Keistraat, Sint-Martens-Latem, Belgium) with the Dice similarity coefficient method.

### Analysis of homologous gene families and phylogenetic construction.

Sequences shorter than 50 amino acids in length were removed to construct a file containing a basic database and a query database. An all-VS-All Blastp analysis was performed with thresholds of 1e−10. Homology between the predicted proteomes was determined with OrthFinder (v2.2.7) (48), and sequences with 70% or more alignment were considered homologous. Gene family clusters were identified by MCL with 1.5 inflation, followed by visualization using homemade Perl scripts.

Phylogenetic analysis of the strains was performed by multiple sequence alignment of single-copy genes based on homologous clusters. The genomic data were aligned using MAFFT (http://mafft.cbrc.jp/alignment/software/), and alignment quality control was conducted using Gblocks (v0.91b) (http://molevol.cmima.csic.es/castresana/Gblocks.html). A maximum-likelihood tree with 1,000 bootstrap replicates was generated using RAxML (v8.2.12) (49) to verify the reliability of the phylogenetic tree branches.

### Variant calling.

Paired reads were mapped to the CRKP HS11286 reference genome (ST11) using Snippy 3.2 (https://github.com/tseemann/snippy). Sequences were excluded if <70% of the genome was identified. Variants were identified across all mapped nonrepetitive sites using SAMtools v1.3.1 (50), and this required a proportion of >75% across all reads and a minimum read depth of ≥ 5 (at least one in each direction).

### String test.

A string test was used to identify the hypermucoviscosity phenotype. Briefly, a bacteriological loop was used to stretch a colony on a blood agar plate. An isolate with a viscous string of >5 mm in length was considered hypermucoviscous (51).

### Serum resistance assay.

A serum resistance assay was performed as described previously (2). Exponential-phase bacterial suspensions adjusted to 1 × 10^6^ CFU/mL were mixed at a 1:3 ratio with healthy human serum and then incubated at 37°C for 2 h. The results were analyzed by comparing the number of CFU before and after 2 h of incubation. The bacteria were considered resistant to normal human serum if at least 90% survived, were considered sensitive to serum if the CFU counts dropped to 1%, and were considered intermediately sensitive if exhibiting 1% to 90% survival of the initial CFU count after 2 h of incubation. Hypervirulent K. pneumoniae strain NTUH-K2044 (a clinical hvKP isolate derived from the National Taiwan University Hospital [52]), which is resistant to killing by serum, and a cKp strain that is sensitive to killing by serum were used as positive and negative controls, respectively.

### Siderophore assays.

For the plate siderophore production assay, stationary-phase iron-chelated cultures (3 mL) were dropped onto modified King B agar plates containing chrome azurol S dye (CAS) solution (Coolaber Technology Co. Ltd., Beijing, China). Siderophore production was indicated by the appearance of opaque golden-yellow halos after overnight incubation at 37°C (17). NTUH-K2044, and a cKp strain were used as positive and negative controls, respectively. Three independent replicates were performed for each assay.

For the relatively quantitative solution-based siderophore production assay, a colony of each strain was inoculated into iron-chelated M9 minimal medium containing Casamino Acids (c-M9-CA) and grown overnight at 37°C with shaking at 180 rpm (53). Then, 100 μL of supernatant from each bacterial suspension was diluted 5-fold in c-M9-CA and added to 100 μL CAS solution in a flat-bottom 96-well plate. After incubation in the dark for 30 min, the absorbance was measured at 630 nm. c-M9-CA plus CAS solution was used as a reference. Siderophore units were calculated as follows: [(*A*_reference_ − *A*_test solution_)/*A*_reference_] × 100%. Each assay was performed in duplicate and repeated twice independently.

### Galleria mellonella infection model.

Galleria mellonella larvae (pathogen-free, 250 to 350 mg) were obtained from Huiyude Biotech Company (Tianjin, China). Ten larvae from each group were injected with 10 μL exponential-phase bacterial suspension (at a concentration of 10^7^ CFU/mL) into the left proleg. NTUH-K2044, and a cKp strain were used as positive and negative controls, respectively. The larvae were then placed in petri dishes and incubated at 37°C in the dark for 72 h to observe mortality rates (6).

### Bacterial growth.

Growth curves of bacterial strains were determined as described before (6). Generally, overnight cultures were diluted 1:1,000 in 30 mL of fresh LB medium and continuously grown at 37°C with shaking at 200 rpm. The cell density was determined every 1 h or 2 h by measuring the optical density at 600 nm (OD_600_).

### Semiquantitative biofilm assay.

The ability of selected K. pneumoniae strains to form biofilms was determined as described previously (54). An overnight bacterial culture was suspended with LB broth at 0.5 McFarland, then was diluted 1:100 and transferred to 96-well plates with 200 μL LB medium. After incubation for 24 h at 37°C, the supernatant was removed, and then each well was washed three times with phosphate-buffered saline. Organisms in the wells were later stained with 1% crystal violet for 30 min at room temperature, and the floating stain was washed off with slow-running water. After drying, the stained biofilm was solubilized with 200 μL of absolute alcohol and quantified by measuring the absorbance at 590 nm. The assay was performed in triplicate.

### Statistical analysis.

SPSS 26.0 (SPSS Inc., Chicago, IL, USA) software was used for analyzing the data. Student's *t* test or Wilcoxon rank sum test was performed for continuous variables. A chi-square test or Fisher’s exact test was performed for categorical variables. ICCs were calculated to analyze consistency between colonizing and infecting isolates. A *P* value of < 0.05 was considered statistically significant.

### Data availability.

The data that support the findings of this study have been deposited with the National Center for Biotechnology Information under Bioproject PRJNA858403.
