# Supplementary material for: Association between Intestinal Colonization and Extraintestinal Infection with Carbapenem-Resistant Klebsiella pneumoniae in Children
Source: Microbiol Spectr. 2023 Mar 14;11(2):e04088-22. doi: 10.1128/spectrum.04088-22 (PMC10100809; doi:10.1128/spectrum.04088-22)
Supplement: Supplemental file 2 — Supplemental material. Download spectrum.04088-22-s0002.pdf, PDF file, 0.4 MB [file spectrum.04088-22-s0002.pdf]

Supplementary Figures:

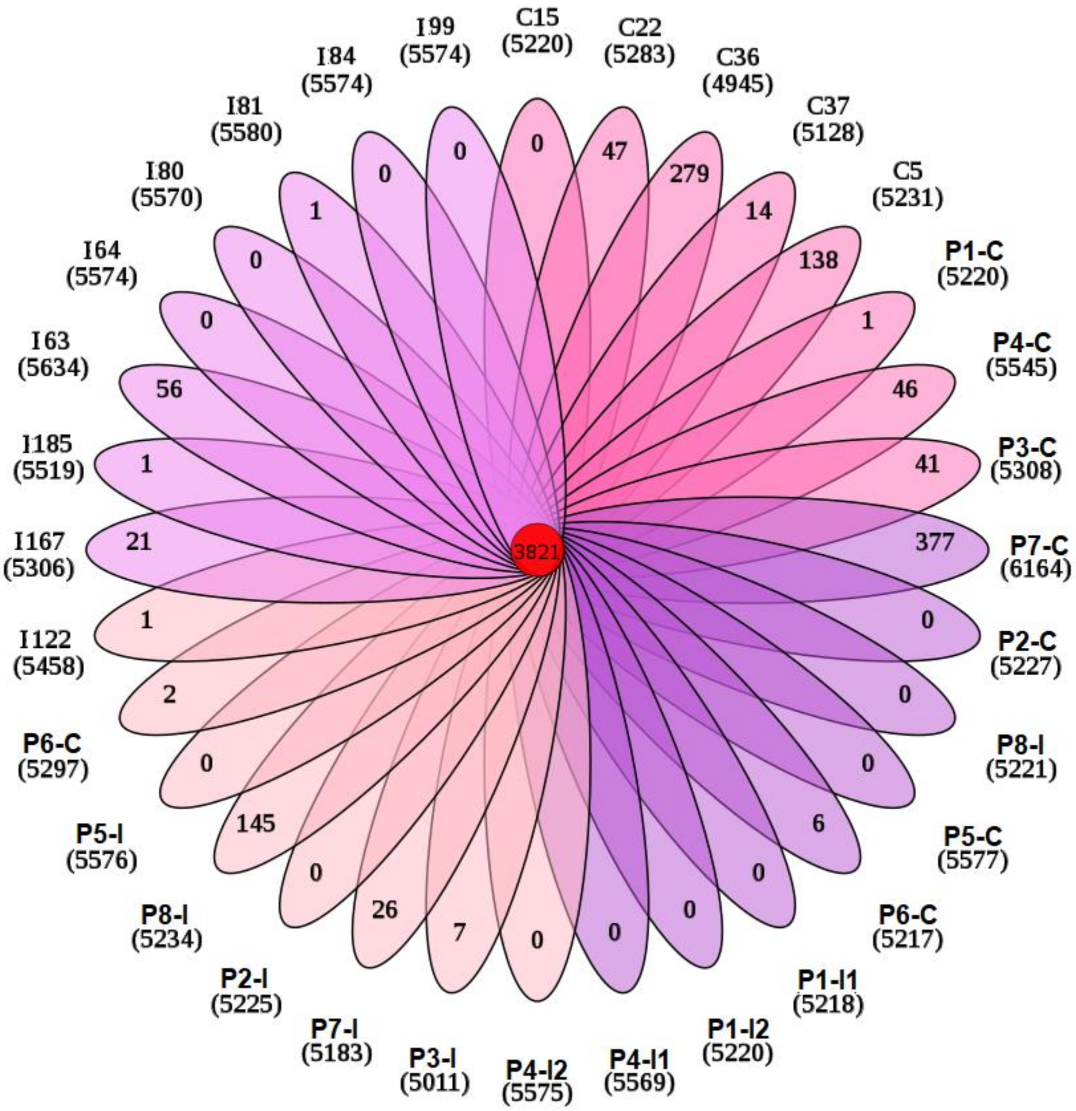

FIGURE S1 Gene family statistics of 32 CRKp strains. The number of genes identified in each strain is shown out of the petals; the number of unique gene families in each strain is shown on the petal tip, while the number of gene families shared by all the 32 strains is shown in the center of the flower.

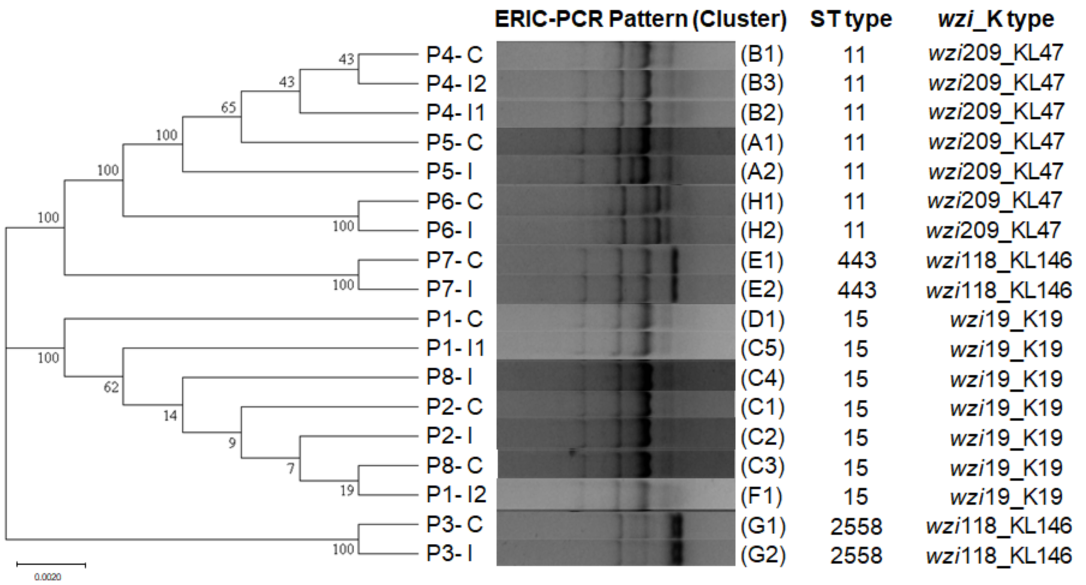

FIGURE S2 Homology analysis based on core genome phylogenetic analysis, ERIC-PCR, MLST, and *wzi* typing of the pairwise colonizing and infecting isolates (n = 18). The phylogenetic relationships were analyzed by MAFFT using multiple sequence alignment of single-copy homologous genes based on maximum-likelihood analysis. The evolutionary analysis for finger printing profiles of Intergenic Consensus-Polymerase Chain Reaction (ERIC-PCR) was performed by BioNumerics software. Abbreviations: P1-8: patient 1-8; C: colonization; I: infection.

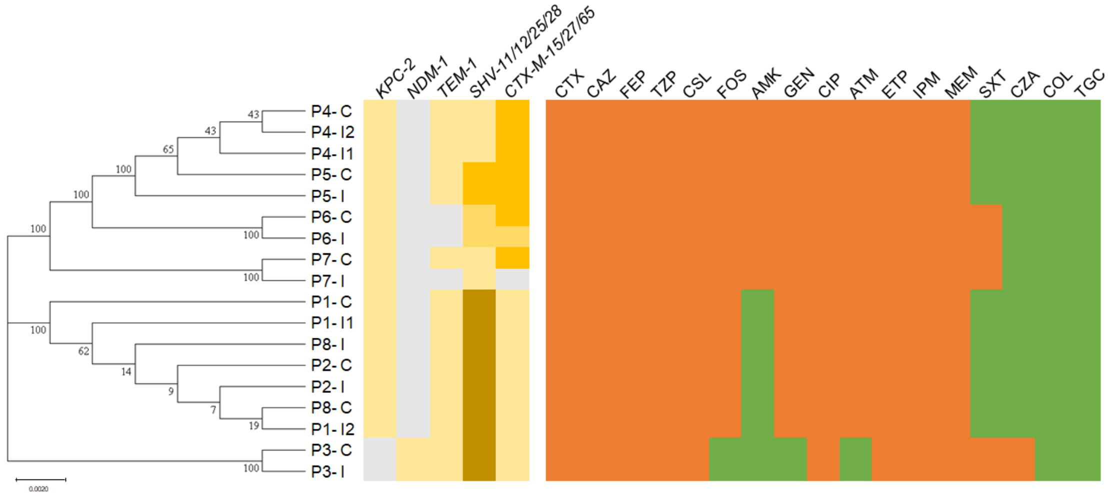

FIGURE S3 Resistance determinants and antimicrobial susceptibility of of the pairwise colonizing and infecting isolates (n = 18). In the left-hand panel, grey indicates that the gene is absent, and all other colors indicate that the gene is present; in the SHV and CTX-M columns, the color intensity increases with the subtyping number in each isolate. In the right-hand panel, orange indicates resistance to drugs, and green indicates susceptibility to drugs. Abbreviations: P1-8: patient 1-8; C: colonization; I: infection. CTX, cefotaxime; CAZ, ceftazidime; FEP, cefepime; TZP, piperacillin/tazobactam; CSL, cefoperazone/sulbactam; FOS, fosfomycin; AMK, amikacin; GEN, gentamicin; CIP, ciprofloxacin; ATM, aztreonam; ETP, ertapenem; IPM, imipenem; MEM, meropenem; SXT, trimethoprim/sulfamethoxazole; CZA, ceftazidime-avibactam; COL, colistin; TGC, tigecycline.
